# *Limosilactobacillus reuteri* regulates gut microbiota and increases the effective metabolite luteolin to inhibit MAPK/STAT3 signaling pathway to alleviate allergic rhinitis

**DOI:** 10.3389/fmicb.2025.1522191

**Published:** 2025-02-24

**Authors:** Mingyan Zhang, Xuewei Sun, Xiang Yu, Li Xu, Xinrui Zhang, Ruonan Zhang, Han Lu, Yujie Wang, Fei Xue, Ting Zhang, Chengliang Tang, Zihan Wu, Zhuohan Zhang, Jin Zhu, Qian Cui, Zhan Yang, You Cheng

**Affiliations:** ^1^Jinling Clinical Medical College, Nanjing University of Chinese Medicine, Nanjing, China; ^2^Huadong Medical Institute of Biotechniques, Nanjing, China; ^3^Department of Otolaryngology-Head and Neck Surgery, Jinling Hospital, Medical School of Nanjing University, Nanjing, China; ^4^School of Medicine and Holistic Integrative Medicine, Nanjing University of Chinese Medicine, Nanjing, China; ^5^Department of Pathogen Biology, Nanjing Medical University, Nanjing, China; ^6^The Second Affiliated Hospital of Nanjing Medical University, Nanjing, China; ^7^Air Force Hospital of Eastern Theater, Nanjing, China

**Keywords:** allergic rhinitis, *Limosilactobacillus reuteri*, gut microbiota, metabolite, luteolin, immune regulation

## Abstract

The global prevalence of allergic rhinitis (AR) remains high, posing challenges due to its chronic nature and propensity for recurrence. Gut microbiota dysbiosis contributes to immune dysregulation, impacting AR pathogenesis. *Limosilactobacillus reuteri* (*L. reuteri*) has great potential in regulating immune function to alleviate AR symptoms. However, the specific active components and mechanisms underlying its therapeutic effects in AR remain incompletely clarified. This study aimed to explore the potential mechanisms of *L. reuteri* and its metabolites in alleviating AR. The AR mouse model was constructed using ovalbumin (OVA). The analysis of hematoxylin–eosin staining (HE staining) and enzyme-linked immunosorbent assay (ELISA) suggested that *L. reuteri* alleviated nasal inflammation, suppressed aberrant Th2 immune responses, and modulated the balance of Treg and Th17 cytokines. The 16S rRNA sequencing and untargeted metabolic analysis revealed that *L. reuteri* restored gut microbiota composition and significantly increased the abundance of *Ligilactobacillus* and the metabolite luteolin (LO). Through ELISA and Western blotting analysis, LO treatment restored the Th1/Th2 and Treg/Th17 cytokine balance and suppressed the MAPK/STAT3 signaling pathway in AR mice. The study highlights LO as a key metabolite contributing to the anti-inflammatory effects of *L. reuteri*, suggesting potential avenues for future therapeutic strategies in AR management.

## 1 Introduction

Allergic rhinitis (AR) is a common chronic inflammatory condition affecting the upper respiratory tract (Bousquet et al., 2020). Over the past few decades, significant changes in environmental exposures and lifestyles have contributed to the increasing prevalence of AR, thereby adversely affecting individuals’ quality of life. It is estimated that approximately 500 million individuals worldwide are affected by AR (Ozdoganoglu and Songu, 2012). The AR prevalence in Asia ranges approximately 5–35%, depending on the method of diagnosis. In Europe, the most recent estimates put AR prevalence at around 25% (Wise et al., 2023). AR is classified as a type I hypersensitivity reaction, triggered by exposure to allergens in susceptible individuals (Bernstein et al., 2016). The production of immunoglobulin E (IgE) and the polarization of T helper type 2 (Th2) cell responses are key pathogenic factors in AR (Okubo et al., 2020). Clinical symptoms typically manifest as episodic and intermittent attacks, which may occur seasonally or persist throughout the year, and commonly include sneezing, rhinorrhea, nasal pruritus, and nasal congestion (Schuler and Montejo, 2019; Sur and Plesa, 2015). AR primarily arises from the secretion of IL-4, IL-5, and IL-13 by Th2 cells, which affects the balance between T helper type 1 (Th1) and Th2 cells, thereby triggering IgE production (Abdel-Gadir et al., 2019). Conversely, Th1 cells mitigate AR symptoms by secreting anti-inflammatory agents such as IFN-*γ*, which suppress the Th2 immune response (Takahashi et al., 2006).

Currently, intranasal corticosteroids are the most effective treatment (Sur and Plesa, 2015). Prolonged use of these medications can lead to side effects such as headache, nasal bleeding, and dryness, which not only diminish quality of life (QOL) but also increase health risks (Liang et al., 2020). The high costs and adverse effects of these drugs are significant. Thus, developing more affordable drugs or treatments with fewer side effects is crucial for helping patients to manage their illnesses and improve their lives.

The intestinal microbiota refers to microorganisms residing in the intestinal tract, where beneficial species such as *Bifidobacterium* and *Lactobacillus* contribute positively to human health. The intestinal microbiota acts as a crucial signaling center, integrating environmental stimuli with genetic and immune signals to significantly influence host metabolism, immunity, and resistance to infection (Thaiss et al., 2016). *Lactobacillus*, one of the earliest discovered probiotics, has been widely used in various foods and dietary supplements (Gangaiah et al., 2022). Studies have shown that *Lactobacillus* can restore the intestinal microenvironment and effectively regulate immune homeostasis (Zhou et al., 2020). A study found that administering *Lactobacillus paracasei* (*LP-33*) for 6 weeks to children with perennial allergic rhinitis was similarly effective to cetirizine, with nearly all children experiencing significant symptom improvement (Ahmed et al., 2019). *Lactobacillus plantarum GUANKE* effectively alleviated AR symptoms by maintaining a balanced ratio of Th1 and Th2 immune cells and regulating chemokine production (Han et al., 2023). *Limosilactobacillus reuteri* (*L. reuteri*) is one of the most extensively studied probiotic strains (Rao et al., 2021). *L. reuteri CCFM1072* and *CCFM1040* were found to reduce airway inflammation in mice with allergic asthma, suppress Th2 and Th17 immune responses, promote Treg response, and enhance gut microbiota diversity and metabolism (Li et al., 2022). Another study showed that *L. reuteri* significantly reduced serum concentrations of IgE and Th2 cytokines while increasing the production of the important microbial metabolite butyric acid by remodeling the intestinal microbial structure (Chen et al., 2018).

However, the precise metabolites and underlying mechanisms through which *L. reuteri* exerts its ameliorative effects on AR remain elusive. The exploration of gut microbiome-metabolite interactions could shed light on the connections between the gut and human health, such as mental health, autoimmune disorders, and chronic inflammation. Based on gut microbiome-metabolite interactions, this study studied the effects of *L. reuteri* and its metabolite luteolin (LO) on allergic rhinitis. This study aims to delve deeper into how *L. reuteri* regulates gut microbiota to modulate immune responses and how associated metabolites contribute to its therapeutic effects on AR. We confirmed that *L. reuteri* alleviates nasal inflammation symptoms, suppresses aberrant Th2 immune responses, balances Treg/Th17 cytokines, and restores gut microbiota composition in AR mice through ELISA and 16S rRNA sequencing analysis. Additionally, untargeted metabolic analysis revealed that *L. reuteri* increases the metabolite luteolin, further enhancing its role in AR.

## 2 Materials and methods

### 2.1 Culture of *Limosilactobacillus reuteri*

The *L. reuteri* strain was obtained from China Center for Type Culture Collection (CCTCC). The strain was cultured in deMan Rogosa Sharpe (MRS) broth at 37°C under anaerobic conditions for 12 to 16 h.

### 2.2 Animals management

Specific-pathogen-free (SPF) BALB/c female mice (6 weeks old, 16–20 g, SiPeiFu Biotechnology Company, Suzhou, China) were housed in an environmentally controlled animal facility. Mice were maintained on a 12-h light/12-h dark cycle to simulate natural circadian rhythms and preserve physiological homeostasis. The temperature indoors ranged from 20 to 26°C, with humidity maintained between 40 and 70% to prevent environmental stress. This animal study protocol was approved by the Ethics Committee of Huadong Medical Institute of Biotechniques (No.2024NKY012).

### 2.3 *Limosilactobacillus reuteri* treatment of the OVA-induced AR mice

The AR mice model was established through ovalbumin (OVA) induction. After a week adaptation period, mice were randomly assigned to three groups (*n* = 6): the negative control (NC) group, the OVA group, and the *L. reuteri* (LR) group. In brief, OVA-induced mice were sensitized via intraperitoneal injection with a mixture of 100 μg ovalbumin (OVA; Sigma, St. Louis, MO) and 4 mg aluminum hydroxide (Solarbio, Beijing, China) in 200 μL PBS on days 1, 3, 5, 7, 9, 11, and 13. Subsequently, mice were intranasally challenged with 10% OVA (10 μL per nasal cavity) daily from day 15 to 21. During OVA induction, mice in the LR group received daily oral administration of 0.2 mL *L. reuteri* suspension containing 1 × 10^9^ colony forming units for 3 weeks. The NC group were gavaged with PBS all the time. After the final OVA challenge, nasal sneezing and nose scratching frequency were recorded for 10 min, concurrent with fecal sample collection.

### 2.4 Luteolin treatment of the OVA-induced AR mice

Mice were randomly assigned to five groups (*n* = 8): the NC group, the OVA group, and three experimental groups receiving different doses of luteolin (5-LO: 5 mg/kg, 10-LO: 10 mg/kg, 20-LO: 20 mg/kg). To induce an allergic response, mice were intraperitoneally injected with a solution containing 100 μg ovalbumin and 4 mg aluminum hydroxide on days 1, 3, 5, 7, 9, 11, and 13. From day 15 to day 28, mice underwent intranasal stimulation with 10% OVA (Dong et al., 2021). During intranasal OVA stimulation, mice in the LO group was administered intraperitoneally luteolin (Merced, California, United States), dissolved in 0.2 mL normal saline, once daily from day 15 to 28. After the final OVA challenge, nose-scratching frequency was observed and recorded for 10 min.

### 2.5 Hematoxylin and eosin staining

After sacrificing the mice, nasal mucosal tissues were fixed in 4% paraformaldehyde (Beyotime, Shanghai, China) to preserve morphology. The fixed tissues underwent ethanol dehydration and paraffin embedding. Paraffin blocks were sectioned into 5 μm thickness using a Minux S700A automatic rotary slicer (RWD Life Sciences, Shenzhen, China). Sections were dewaxed in xylene, rehydrated in ethanol, and stained with hematoxylin (Biyuntian, Shanghai, China) for 20–30 s, followed by a distilled water rinse. Excess stain was removed with hydrochloric acid ethanol for 5 s (Pician, Shanghai, China), and sections were briefly treated with ammonia for 5 s (Solarbio, Beijing, China) to enhance nuclear clarity. Following eosin staining (20 s), sections were dehydrated, cleared with xylene, and examined under a CX23 microscope at 400x magnification (Olympus, Tokyo, Japan). Microscopic analysis enabled the observation of cell morphology, structure, and nasal mucosal lesions.

### 2.6 Enzyme-linked immunosorbent assay

ELISA was performed to quantify OVA-sIgE in serum, along with IL-4, IL-5, IL-13, IFN-*γ*, IL-17, IL-10, RORγt, and Foxp3 levels in both nasal lavage fluid (NALF) and serum samples (Lapuda, Nanjing, China). Samples were preprocessed for uniformity and stability before being adding to 96-well plates coated with specific antibodies. Biotinlabeled secondary antibodies were applied, followed by further incubation and washing steps. A streptavidin-HRP conjugate was added to bind biotin and introduce HRP enzymatic activity. Addition of a chromogenic substrate initiated an HRP-catalyzed oxidation reaction, generating colored products whose absorbance at 450 nm was measured using a microplate reader (Promega, Madison, WI). Antigen concentrations were determined from a standard curve.

### 2.7 16S rRNA sequencing analysis

Fecal samples were collected and subjected to DNA extraction using a Fast DNA Spin Kit (MP Biomedicals, Santa Ana, CA, United States). The hypervariable region V3-V4 of the bacterial 16S rRNA gene were amplified with primer pairs 338F: 5′- ACTCCTAC GGGAGGCAGCA-3′ and 806R: 5′- GGACTACHVGGGTWTCT AAT-3′. PCR products were checked on agarose gel and purified through the Omega DNA purification kit (Omega Inc., Norcross, GA, United States). The purified PCR products were collected and the paired ends (2 × 250 bp) was performed on the Illumina Novaseq 6,000 platform (Fang et al., 2022). Identification and removal of primer sequences was process by Cutadapt (Bolger et al., 2014). PE reads obtained from previous steps were assembled by USEARCH and followed by chimera removal using UCHIME. The high-quality reads generated from above steps were used in the following analysis. Sequences with similarity >97% were clustered into the same operational taxonomic unit (OTU) by USEARCH and the OTUs conuts less than 2 in all samples were filtered (Edgar, 2013). Clean reads then were conducted on feature classification to output an ASVs (amplicon sequence variants) by dada2, and the ASVs counts less than 2 in all samples were filtered. Taxonomy annotation of the OTUs was performed based on the Naive Bayes classifier in QIIME2 using the SILVA database with a confidence threshold of 70% (Quast et al., 2013). The Alpha diversity was calculated and displayed by the QIIME2 and R software, respectively. Beta diversity was determined to evaluate the degree of similarity of microbial communities from different samples using QIIME. Principal coordinate analysis (PCoA) and heatmaps were used to analyze the beta diversity. Furthermore, we employed linear discriminant analysis effect size (LEfSe) to test the significant taxonomic difference among group. A logarithmic LDA score of 4.0 was set as the threshold for discriminative features (Segata et al., 2011).

### 2.8 Untargeted and targeted metabolic analysis

This study employed untargeted metabolic analysis using LC/MS (Mir et al., 2015), which included a Waters Acquity I-Class PLUS liquid chromatograph coupled with a Waters Xevo G2-XS QTOF mass spectrometer. A Waters Acquity UPLC HSS T3 column (1.8 μm, 2.1 × 100 mm) was utilized, with mobile phase A composed of 0.1% formic acid in aqueous solution and mobile phase B composed of 0.1% formic acid in acetonitrile, for both positive and negative ion modes. Data acquisition was performed in MSe mode on the Waters Xevo G2-XS QTOF mass spectrometer controlled by MassLynx V4.2 software. Compound classification and pathway information were obtained from databases such as KEGG, HMDB, and lipid maps. Differential analysis was conducted using grouping information, followed by T-test analysis to determine compound significance (*p*-value).

For targeted metabolism investigation of flavones in supernatants from *L. reuteri* cultures, metabolites were extracted and analyzed using a Waters ACQUITY I-Class ultra-performance liquid chromatograph equipped with an ACQUITY UPLC HSS T3 column (100 mm × 2.1 mm, 1.8 μm particle size, Waters). Prior to UHPLC–MS/MS analysis, standard solutions of target compounds were introduced (Liu et al., 2013). Multiple parent ion-daughter ion pairs were optimized for MRM parameters, selecting the most intense ion pair for quantitative analysis and others for qualitative analysis of target compounds.

### 2.9 Western blotting

WB was used to assess p-STAT3 (1:1000, ab68153), and STAT3 (1:1000, ab267373), and MAPK signaling pathway-associated proteins including p38 (1:1000, #9212), p-p38 (1:1000, #4511). Nasal mucosa samples were weighed and homogenized, and proteins were extracted using a total protein extraction kit (Beyotime, Shanghai, China). Protein concentration was determined using the BCA assay (Vazyme, Nanjing, China). The samples were then loaded into a 10% SDS-PAGE gel for separation and subsequently transferred to a polyvinylidene fluoride (PVDF) membrane. The membranes were blocked with 5% skim milk at room temperature for 1 h, then incubated. Primary antibodies, diluted in TBST at recommended concentrations, were incubated with the membranes overnight at 4°C. After washing with TBST, the membranes were incubated with secondary antibodies at room temperature for 1 h. Protein bands were visualized using a detection reagent (Thermo Fisher Scientific, Waltham, MA, United States), and Image J software was used to quantify band intensity.

### 2.10 Statistical analysis

In this study, measurement data described as means ± standard deviation underwent statistical analyses using GraphPad Prism 9.0 (GraphPad Software, La Jolla, CA). For the comparison of two sets of data, an independent sample t-test was used; whereas for the comparison between multiple groups, a one-way analysis of variance (one-way ANOVA) was performed. In all statistical analyses, a *p*-value less than 0.05 was used as the criterion for statistically significant differences. * *p* < 0.05, ** *p* < 0.01, *** *p* < 0.001, and **** *p* < 0.0001.

## 3 Results

### 3.1 *Limosilactobacillus reuteri* alleviated pathological symptoms in OVA-induced AR mice

**Figure 1:**
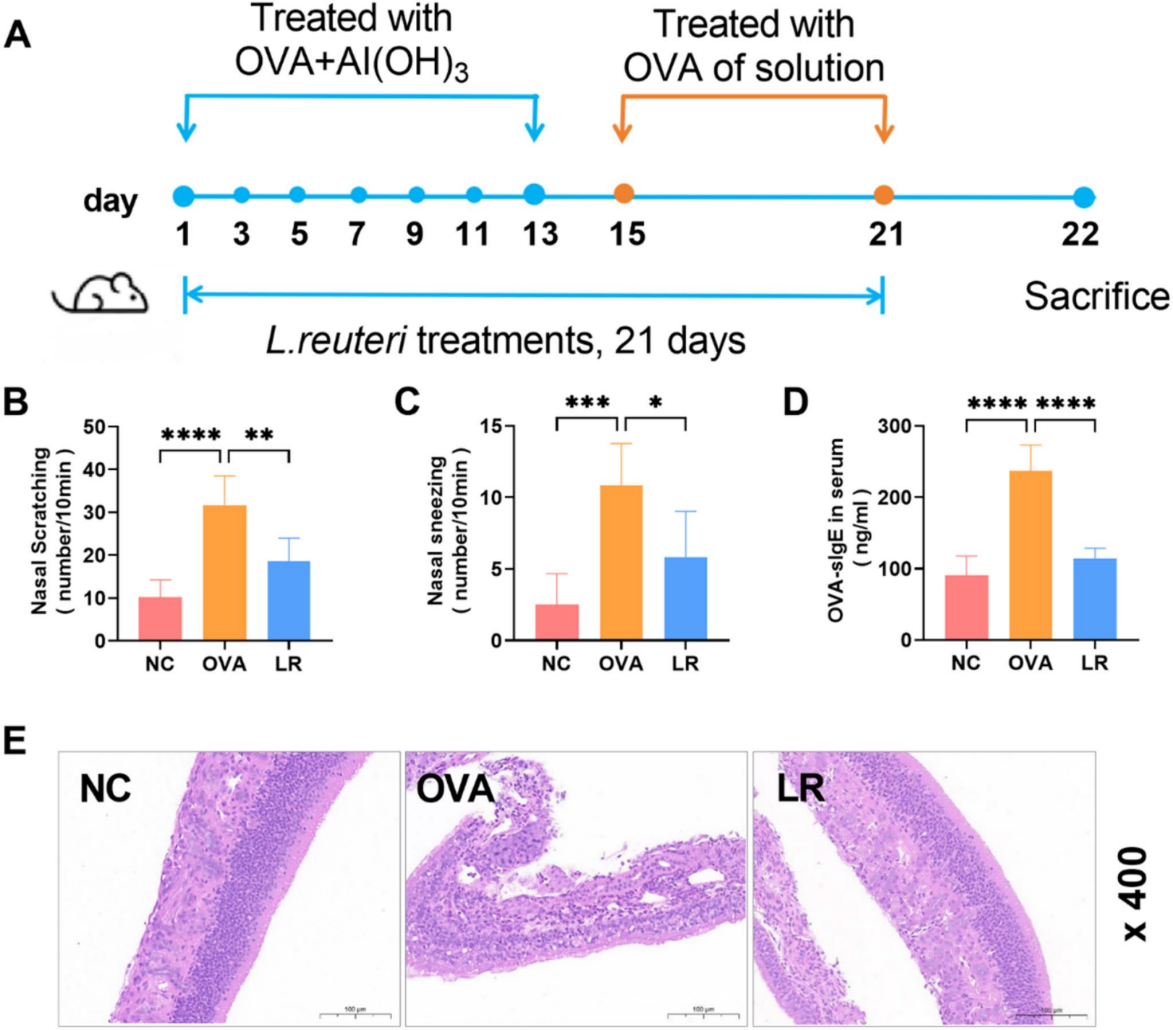
*L. reuteri* alleviated pathological symptoms in OVA-induced AR mice. **(A)** The experimental design of OVA-induced AR mice with dietary supplement of *L. reuteri*. **(B,C)** The frequency of nasal scratching and sneezing. **(D)** The serum level of OVA-sIgE was measured through ELISA. **(E)** HE staining showed the inflammatory cell infiltration of the nasal mucosal tissue (magnification: ×400). Data were presented as means ± SD. Results were analyzed by one-way ANOVA, * *p* < 0.05, ** *p* < 0.01, *** *p* < 0.001, **** *p* < 0.0001. *n* = 6 for each group. NC, negative control; OVA, ovalbumin; LR, *L. reuteri*; AR, allergic rhinitis; ELISA, enzyme-linked immunosorbent assay; HE, hematoxylin–eosin.

To assess the impact of *L. reuteri* strains on the pathological manifestations of AR, a model was established through the induction of OVA. The experimental design was depicted in Figure 1A. The OVA group exhibited significant rhinitis symptoms characterized by frequent nasal scratching and sneezing compared to the NC group. However, these symptoms were reduced in the LR group (Figures 1B,C). Compared to the NC group, the OVA-sIgE serum level in the OVA group exhibited a statistically significant elevation (*p* < 0.0001). Notably, in the LR group, a marked decrease in the OVA-sIgE serum level was observed (Figures 1D, *p* < 0.0001). Moreover, in order to assess pathological changes in mice, the nasal mucosal tissues were subjected to HE staining. The OVA treatment resulted in a significant inflammatory cell infiltration in the nasal cavity of mice, compared to the NC group. *L. reuteri* strain treatments reduced inflammatory cell infiltration and tissue edema compared to the OVA group (Figure 1E).

### 3.2 *Limosilactobacillus reuteri* balanced Th1/Th2 and Treg/Th17 cytokine levels in OVA-induced AR mice

**Figure 2:**
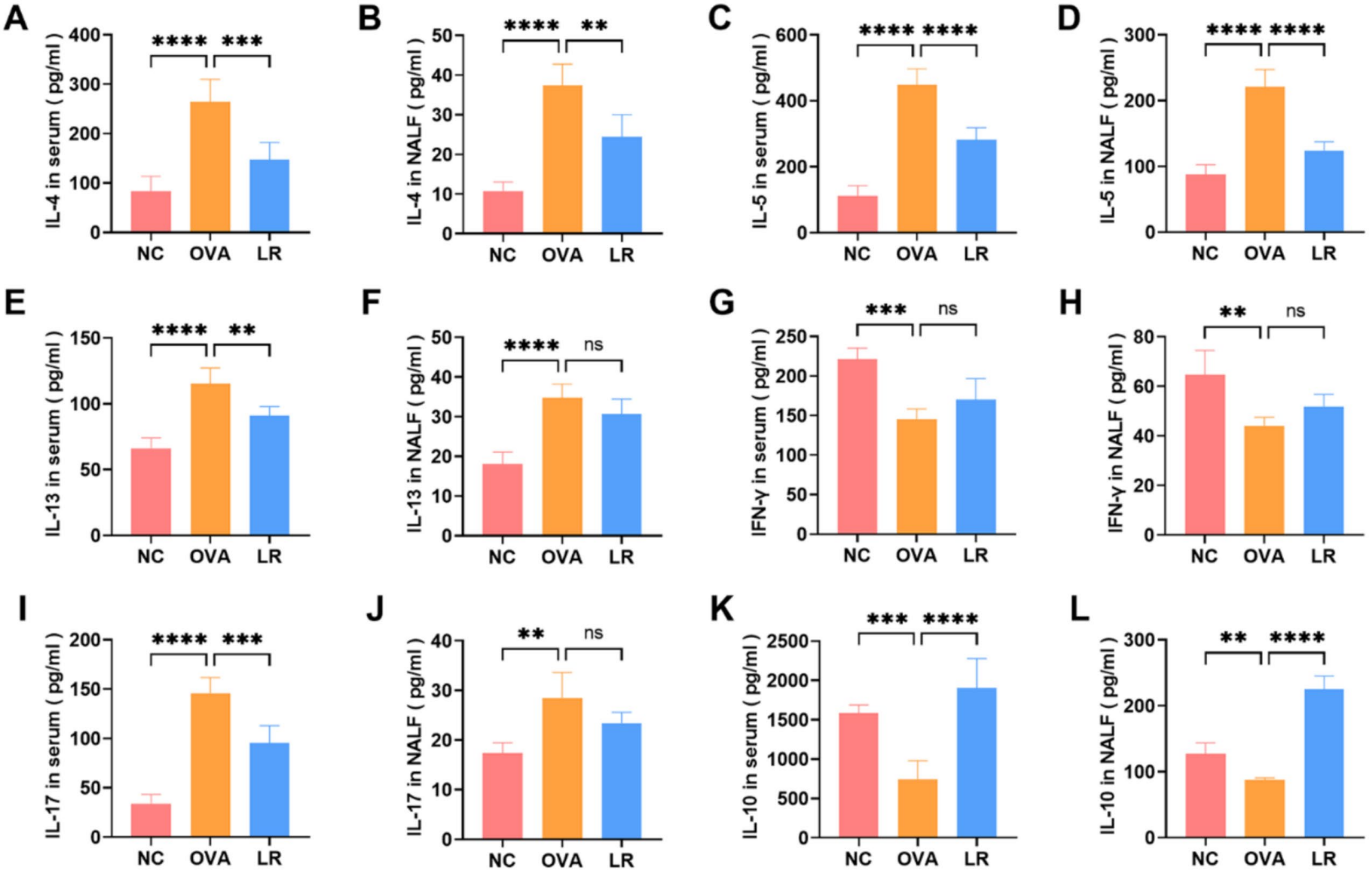
*L. reuteri* balanced Th1/Th2 and Treg/Th17 cytokine levels in OVA-induced AR mice. **(A–F)** The levels of IL-4, IL-5, IL-13 in both serum and NALF. **(G,H)** The level of IFN-*γ* was measured through ELISA. **(I,J)** The level of IL-17 was significantly decreased in the LR group. **(K,L)** The level of IL-10 was significantly increased in the LR group. Data were presented as means±SD. Results were analyzed by one-way ANOVA; ns: not significant, **p < 0.01; ***p < 0.001; ****p < 0.0001. NC, negative control; OVA, ovalbumin; LR, *L. reuteri*; AR, allergic rhinitis; NALF, nasal lavage fluid; ELISA, enzyme-linked immunosorbent assay.

To assess the immunomodulatory impact of *L. reuteri* administration in mice, we evaluated whether the treatment with *L. reuteri* modulates the Th1/Th2 and Treg/Th17 immune responses in the AR mice. Obviously, the OVA group exhibited significantly elevated levels of IL-4, IL-5, IL-13 (indicators of Th2 cell activity) in both serum and NALF compared to the NC group (*p* < 0.0001). In the LR group, these cytokine levels were significantly reduced (Figures 2A–F). In the OVA group, the level of IFN-*γ* (an indicator of Th1 cell activity) was significantly reduced both in the serum and NALF compared to the NC group, but was not significantly increased in the LR group (Figures 2G,H). The level of IL-17 (an indicator of Th17 cell activity) was significantly increased both in the serum and NALF in the OVA group compared to the NC group and significantly decreased in the serum in the LR group (Figures 2I,J). As depicted in Figures 2K,L, the level of IL-10 (an indicator of Treg cell activity) in the OVA group was significantly decreased compared to the NC group (*p* < 0.01). Furthermore, a marked elevation of IL-10 was observed in the LR group, reaching a highly significant level (*p* < 0.0001).

### 3.3 Gut microbiota changed and associated with immune response

**Figure 3:**
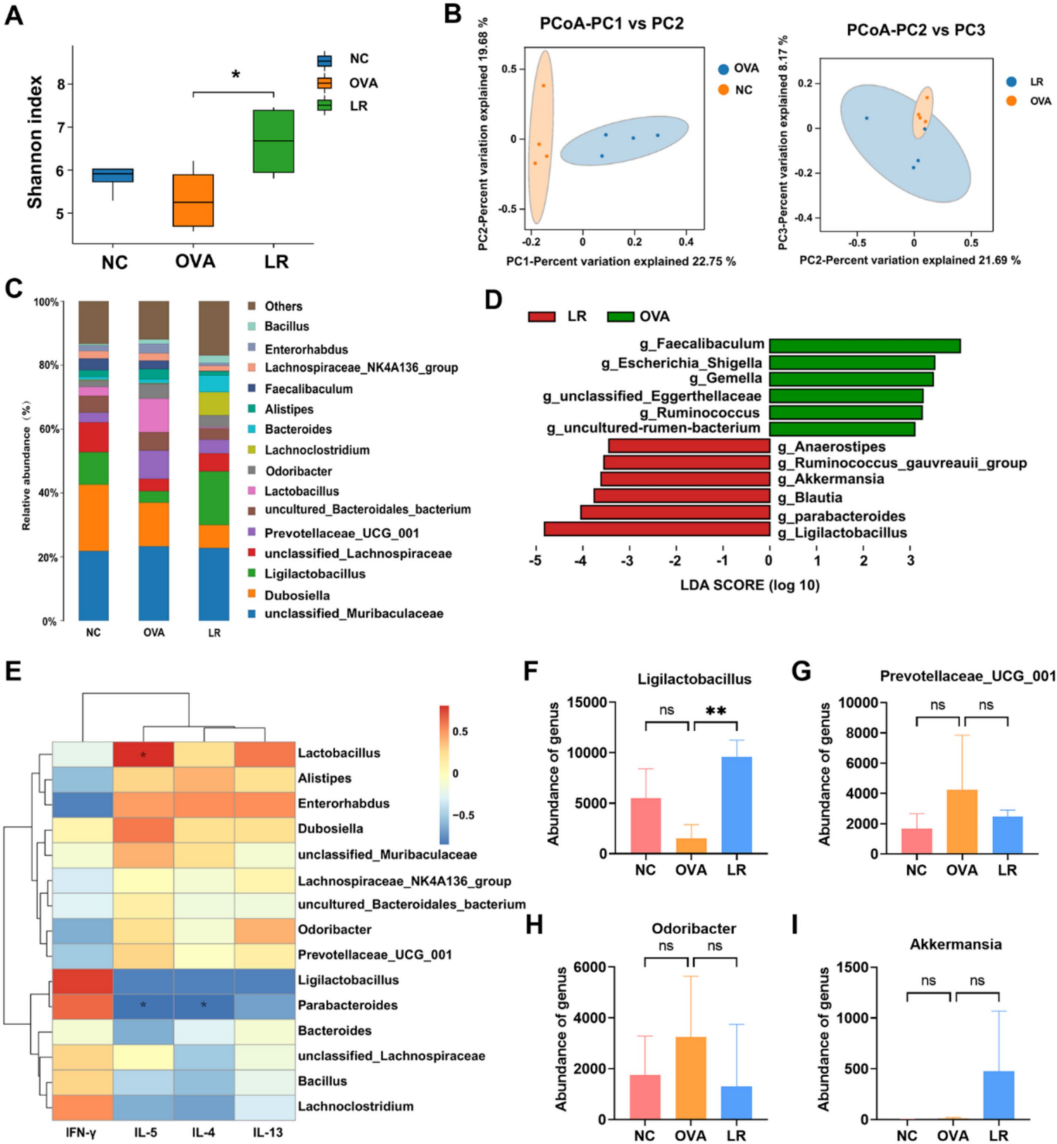
Gut microbiota changed and associated with immune response. **(A)** The Shannon index indicated the change of alpha diversity in OVA-induced AR mice. **(B)** PCoA analysis indicated the change of beta diversity in OVA-induced AR mice. **(C)** The composition of species abundance between the groups at the genus level. **(D)** The LefSe analysis assessed the species abundance composition differences between groups. **(E)** The correlation between different bacteria genera and Th1 and Th2 cytokine levels between the OVA and LR groups. **(F–I)** The genus-level abundance of change of *Ligilactobacillus*, *Prevotellaceae_UCG_001*, *Odoribacter* and *Akkermansia*. Data were presented as means ± SD. Results were analyzed by one-way ANOVA; ns, not significant, ** *p* < 0.01. NC, negative control; OVA, ovalbumin; LR, *L. reuteri*; AR, allergic rhinitis; PCoA, principal coordinates analysis; LEfSe, linear discriminant analysis effect size.

To explore the differences of gut microbiota among the NC, OVA, and LR groups, a comprehensive analysis was conducted using 16S rRNA sequencing analysis on mouse fecal samples. The alpha diversity was assessed using the Shannon index to evaluate species diversity within each group. Compared to the NC group, the Shannon index was reduced in the OVA group. However, there was a notable increase in the Shannon index in the LR group (Figure 3A). The beta diversity analysis was employed to compare the differences in microbial community composition and structure. The PCoA revealed significant differences in microbial community composition between the NC and OVA groups. Additionally, microbial composition also varied between the OVA and LR groups (Figure 3B,). Figure 3C showed the composition of species abundance between the groups at the genus level. The LefSe further evaluated the differences in species abundance between groups to identify statistically significant biomarkers. We identified differential bacteria, such as *Ligilactobacillus*, *Blautia*, *Parabacteroides*, and *Akkermansia* (Figure 3D). Furthermore, the correlation heatmap was created to assess the relationship between different bacterial genera and changes in Th1 and Th2 cytokine levels between the OVA and LR groups. We found that *Prevotellaceae_UCG_001* and *Odoribacter* were positively correlated with IL-4, IL-5, IL-13 cytokines, whereas a positive correlation between the *Ligilactobacillus* and IFN-*γ* cytokines (Figure 3E). The analysis of the relative abundance of individual bacterial genera revealed that supplementation with *L. reuteri* significantly increased the abundance of *Ligilactobacillus* (Figures 3F, *p* < 0.01). This increased abundance of *Ligilactobacillus* in the LR group was strongly positively associated with Th1 cytokines. In contrast, the genus-level abundance of *Prevotellaceae_UCG_001* and *Odoribacter* was decreased in the LR group (Figures 3G,H). The increased abundance of these bacteria in the OVA group was strongly positively associated with Th2 cytokines. However, the abundance of *Akkermansia* increased almost exclusively in the LR group (Figure 3I).

### 3.4 *Limosilactobacillus reuteri* increased production of metabolite luteolin in OVA-induced AR mice

**Figure 4:**
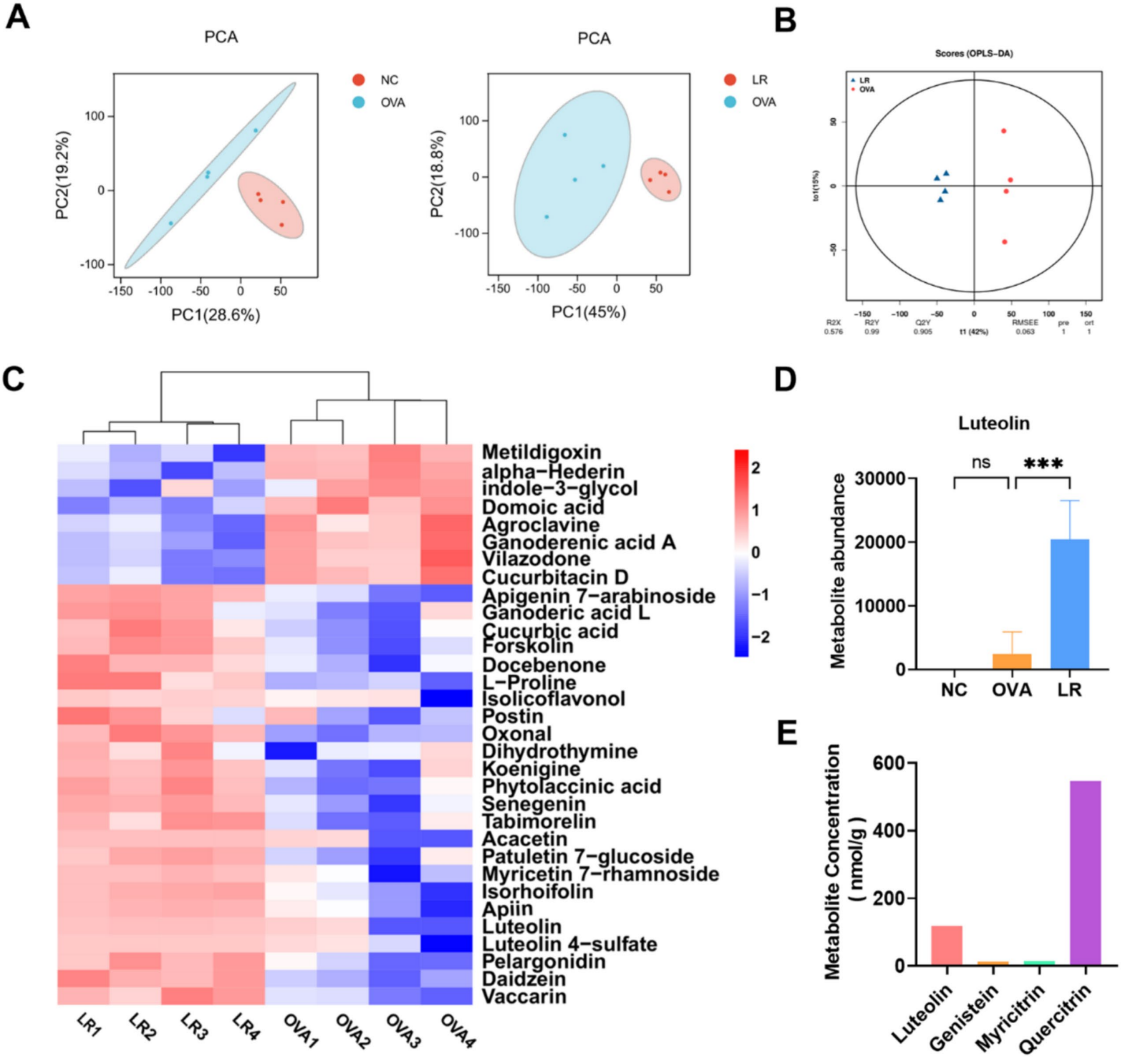
*L. reuteri* increased production of metabolite luteolin in OVA-induced AR mice. **(A)** The PCA analysis indicated differences in metabolic composition among all groups. **(B)** The LR group was considered a valid model for screening differential metabolites compared to the OVA group. **(C)** The differential metabolites in the OVA and LR group, such as Luteolin. **(D)** The abundance of the differential metabolite luteolin. **(E)** A small amount of luteolin was produced in the *L. reuteri* supernatant. Data were presented as means ± SD. Results were analyzed by one-way ANOVA; ns, not significant, *** *p* < 0.001. NC, negative control; OVA, ovalbumin; LR, *L. reuteri*; AR, allergic rhinitis; PCA, principal component analysis.

To further explore the effective substances of *L. reuteri* treatment for alleviating AR symptoms, we conducted an untargeted metabolic analysis on mouse fecal samples. The principal component analysis (PCA) was performed to assess the variation between different groups. As shown in Figure 4A, the metabolite profiles of the NC and OVA groups were significantly different, and there was also a notable variation between the OVA and LR groups. Through orthogonal projections to latent structures-discriminant analysis (OPLS-DA), the LR group was identified as a valid model for screening differential metabolites compared to the OVA group (Figure 4B). We created the hierarchical clustering heatmap of the differential metabolites and observed clear differences in flavonoid compounds between the OVA and LR groups, including luteolin, vaccarin, daidzein, pelargonidin, and senegenin (Figure 4C). The abundance of the differential metabolite luteolin between the LR and OVA groups was significantly increased in the LR group (Figures 4D, *p* < 0.001). Additionally, we performed a pure culture of *L. reuteri* and conducted flavonoids-targeted metabolic analysis on the supernatant of *L. reuteri* culture medium, revealing the production of a small amount of luteolin (Figure 4E).

### 3.5 Luteolin improved pathological symptoms in OVA-induced AR mice

**Figure 5:**
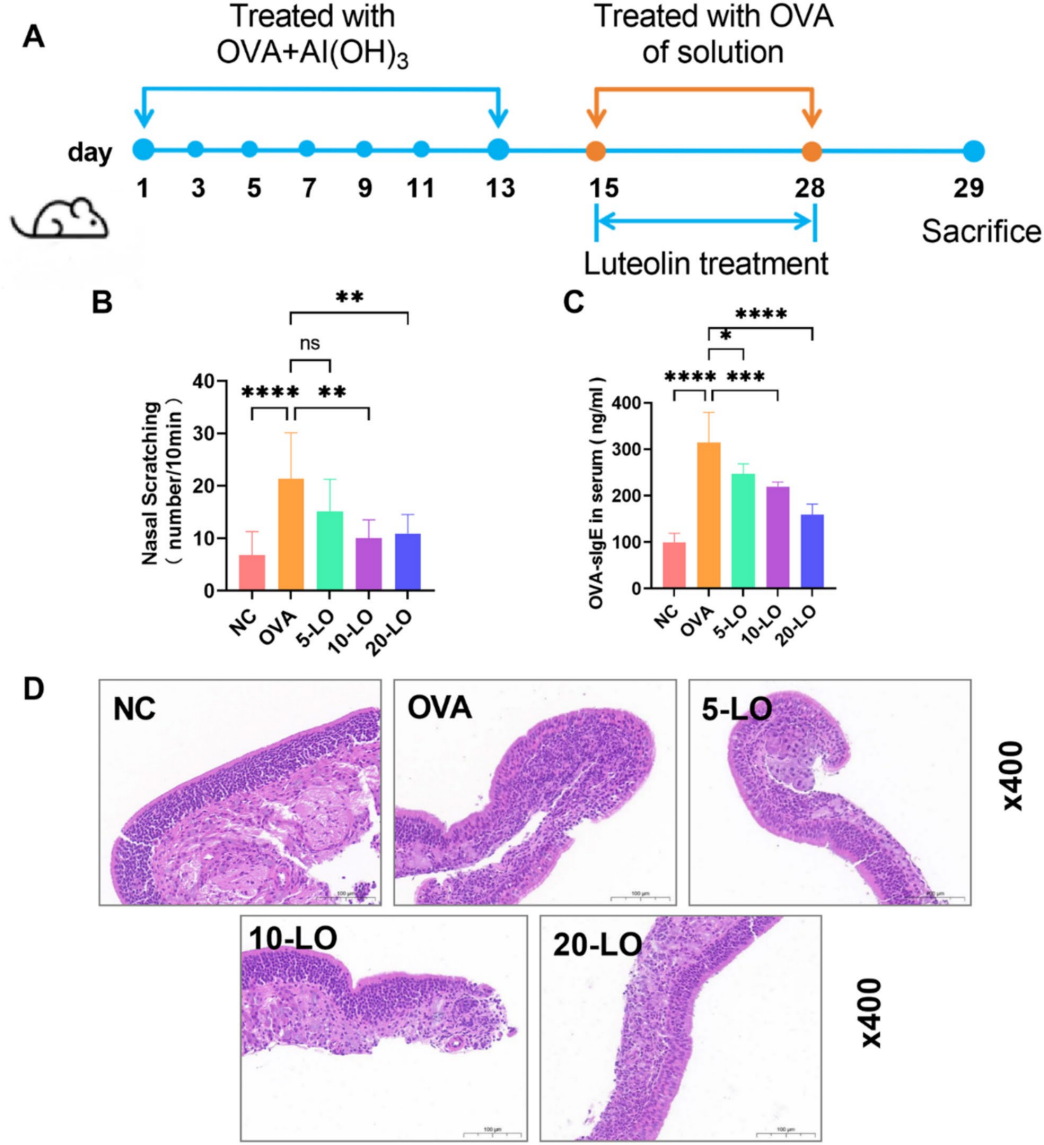
Luteolin improved pathological symptoms in OVA-induced AR mice. **(A)** The experimental design of OVA-induced AR mice with LO treatment. **(B)** The frequency of nasal scratching. **(C)** The serum level of OVA-sIgE was measured through ELISA. **(D)** HE staining showed the inflammatory cell infiltration of the nasal mucosal tissue (magnification: ×400). Data were presented as means ± SD. Results were analyzed by one-way ANOVA, ns, not significant, *p < 0.05; **p < 0.01; ***p < 0.001; ****p < 0.0001. *n* = 8 for each group. NC, negative control; OVA, ovalbumin; LO, luteolin; AR, allergic rhinitis; ELISA, enzyme-linked immunosorbent assay; HE, hematoxylin–eosin.

Figure 5A illustrated the effects of LO treatment in a mouse model of AR. The mice in the OVA group exhibited a significantly higher frequency of nose scratching compared to the NC group. However, the frequency of nose scratching was reduced in the 10-LO and 20-LO groups (Figure 5B, *p* < 0.01). Additionally, mice exposed to OVA showed a significantly elevated serum level of OVA-sIgE in the OVA group. Luteolin treatment effectively ameliorated OVA-induced upregulation of OVA-sIgE, with the most pronounced reduction observed in the 20-LO group (Figure 5C, *p* < 0.0001). HE staining revealed that OVA treatment caused epithelial cell shedding and increased inflammatory cell infiltration in the nasal mucosa, effects which were mitigated by LO treatment (Figure 5D).

### 3.6 Luteolin balanced Th1/Th2 and Treg/Th17 cell cytokine in OVA-induced AR mice

**Figure 6:**
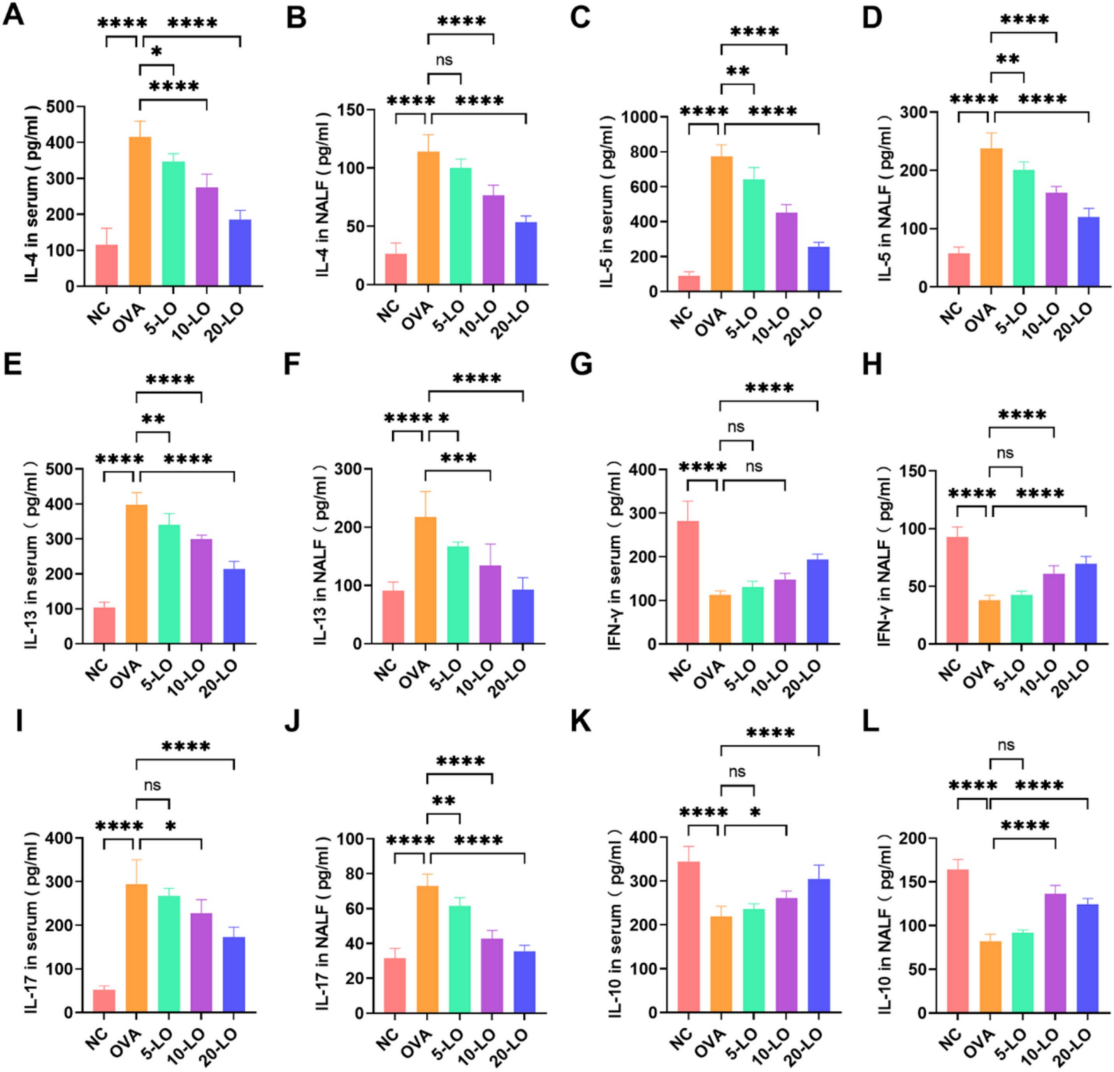
Luteolin balanced Th1/Th2 and Treg/Th17 cell cytokine in OVA-induced AR mice. **(A–F)** The levels of IL-4, IL-5, IL-13 in both serum and NALF. **(G,H)** The level of IFN-γ in serum and NALF was significantly increased in the LO-20 group. **(I,J)** The level of IL-17 was significantly decreased in the LO-20 group. **(K,L)** The level of IL-10 was significantly increased in the LO-20 group. Data were presented as means ± SD. Results were analyzed by one-way ANOVA; ns, not significant, *p < 0.05; **p < 0.01; ***p < 0.001; ****p < 0.0001. NC, negative control; OVA, ovalbumin; LO, luteolin; AR, allergic rhinitis; NALF, Nasal Lavage Fluid.

In the OVA group, we observed a significant increase in the concentrations of IL-4, IL-5, and IL-13 in both NALF and serum compared to the NC group (*p* < 0.0001). In contrast, the levels of these cytokines in the LO-5, LO-10, and LO-20 groups were markedly lower than those in the OVA group (Figures 6A–F). The serum level of IFN-*γ* was not significantly elevated in the LO-5 and LO-10 groups, but was significantly higher in the LO-20 group compared to the OVA group (Figure 6G). Additionally, the level of IFN-γ in NALF was significantly increased in the 10-LO and LO-20 groups (Figure 6H). Th2 cytokine levels decreased significantly with increasing doses of luteolin, whereas Th1 cytokines increased notably at higher doses. Notably, in the OVA group, IL-17 levels were significantly elevated in both serum and NALF compared to the NC group. This increase was significantly reduced in the LO-20 group (Figures 6I,J). Moreover, IL-10 levels in serum and NALF were significantly higher in the LO-20 group (Figures 6K,L).

### 3.7 Luteolin suppressed MAPK/STAT3 signaling pathway

**Figure 7:**
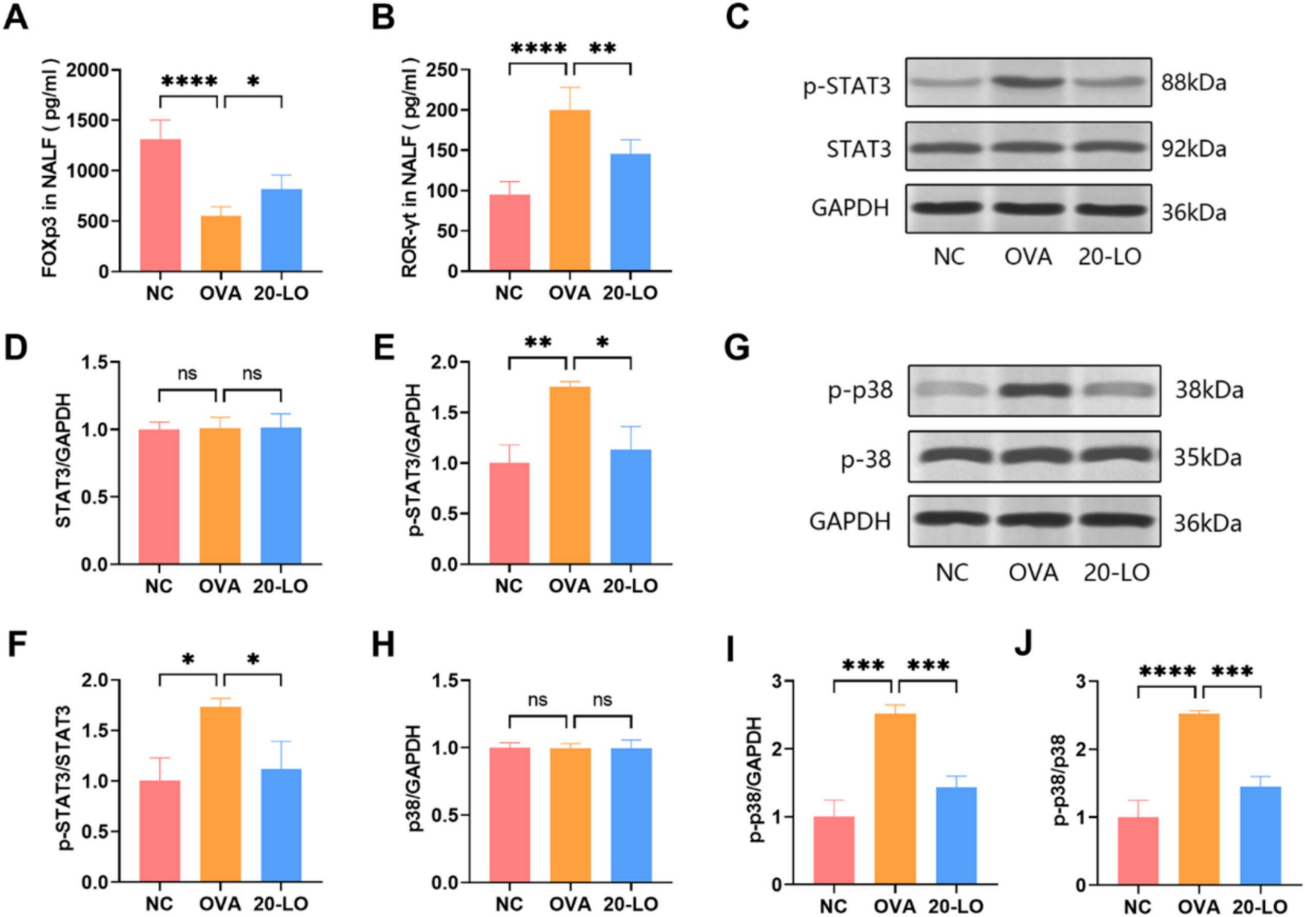
Luteolin suppressed MAPK/STAT3 signaling pathway. **(A)** The Foxp3 levels were increased in NALF in the 20-LO group. **(B)** The NALF level of RORγt was measured through ELISA after LO treatment. **(C–F)** Western blot was used to examine the proteins (STAT3, p-STAT3) in the nasal mucosa of OVA-induced AR mice after LO treatment. GAPDH was the loading control. **(G–I)** Western blot was investigated protein expression along the MAPK signaling pathway (p38, p-p38). **(J)** The p-p38/p38 ratios in the 20-LO groups was decreased. Data were presented as means ± SD. Results were analyzed by one-way ANOVA; ns: not significant, *p < 0.05; **p < 0.01; ***p < 0.001; ****p < 0.0001. NC, negative control; OVA, ovalbumin; LO, luteolin; Foxp3, forkhead box P3; RORγt, retinoic acid receptor-related orphan nuclear receptor; NALF, Nasal Lavage Fluid; ELISA, enzyme-linked immunosorbent assay; AR, allergic rhinitis; GAPDH, glyceralde-hyde-3-phosphate dehydrogenase.

RORγt is a major transcription factor for IL-17, while Foxp3 is a key marker of Treg cells. To determine if RORγt promotes Th17 polarization in AR mice, we measured its concentration in NALF by ELISA. In the OVA group, Foxp3 levels were significantly reduced in NALF, whereas they were increased in the 20-LO group (Figure 7A). RORγt levels in the OVA groups were notably higher compared to the NC group, and the increase was significantly reversed in the 20-LO group (Figure 7B). To further investigate the mechanisms, we assessed the expression of the Th17-related STAT3 signaling pathway by measuring p-STAT3 and STAT3 levels. The western blotting analysis revealed that p-STAT3 protein expression in the nasal mucosa was significantly higher in the OVA group compared to the NC group (Figure 7C). LO treatment effectively reduced p-STAT3 protein expression (Figures 7D,E). The p-STAT3/STAT3 ratios in the 20-LO group showed a significant decrease (Figure 7F). This suggests that the OVA-induced AR model presented an activated STAT3 signaling pathway. Next, we examined the inhibitory effects of luteolin on the MAPK signaling pathway activation. Western blot analysis was used to investigate protein expression within the MAPK signaling pathway, which is involved in the inflammatory response. Phosphorylation of p38 was significantly higher in the nasal mucosa of the OVA group compared to the NC group and was notably reduced in the 20-LO group (Figures 7G–I). Additionally, the p-p38/p38 ratios in the 20-LO group showed a significant decrease (Figure 7J).

## 4 Discussion

This study comprehensively demonstrates that treatment with *L. reuteri* effectively alleviates the symptoms of AR. It not only rectifies immune dysregulation but also modulates the imbalance of the intestinal microbiota. Notably, luteolin, a metabolite identified during the *L. reuteri* treatment process in AR mice, has been found to play a significant role. This highlights the importance of the gut - microbiota - immune axis in the pathogenesis of AR and the potential of microbiota - derived metabolites in treating allergic diseases.

Targeting the gut microbiota may serve as a promising preventive and therapeutic strategy for AR. The pathogenesis of AR remains highly complex and not fully understood. Although multiple studies have demonstrated the significant potential of *L. reuteri* in managing allergic disorders (Chen et al., 2018; Fang et al., 2022), the precise underlying mechanisms are yet to be fully elucidated.

Traditional drug treatment for AR, such as antihistamines and glucocorticoids, can alleviate symptoms, but there are different degrees of side effects, long-term use may also affect the immune balance of the body (Chen et al., 2020; Ren et al., 2017). Most previous studies focused on the pathogenesis of AR. For example, Mengze Ding et al. demonstrated Mahuang Fuzi Xixin decoction suppressed nasal epithelial pyroptosis by inhibiting the NLRP3/Caspase-1/GSDMD-N signaling pathway (Ding et al., 2024).

However, this study explores a new biological therapy path from the regulation of human microecology. This study integrates microbiome and metabolomics analysis, demonstrating that dietary supplementation with *L. reuteri* can modulate the immune response, reshape gut microflora composition, and increase the production of the metabolite luteolin, thereby alleviating allergic nasal inflammation and AR symptoms.

*L. reuteri* strains show promise in alleviating AR symptoms through immune modulation. Multiple studies have demonstrated a beneficial effect of *L. reuteri* on allergic diseases. In a study of multiple probiotics on asthma, *L. reuteri* was the most effective in reducing airway inflammation, total IgE and Th2-associated pro-inflammatory cytokines, and regulating specific microbial genera to prevent asthma (Choi et al., 2021). In an animal experiment, *L. reuteri Fn041*, isolated from breast milk, adjusted Th1/Th2 cytokine ratios, promoted Treg cell production, and altered gut microbiota by increasing the abundance of *Lactobacillus* and *Akkermansia* abundance in atopic dermatitis mice (Zhao et al., 2022). In our study, we found *L. reuteri* treatment enhanced gut microbiota diversity and increased the abundance of certain beneficial bacteria, such as *Ligilactobacillus*. *Ligilactobacillus*, a gastrointestinal lactobacillus and probiotic, which likely plays a role in *L. reuteri*-treated organisms. Additionally, we found the abundance of *Akkermansia* genus increased only in the LR group, consistent with previous reports.

While many studies have demonstrated the efficacy of probiotics in alleviating AR, few have investigated the specific metabolites involved in related mechanisms. Changes in specific microbiota can significantly impact gut microbiota metabolism. Lactobacillus metabolites are rich and diverse, including short-chain fatty acids, bactericins, polysaccharides, and so on (Liu et al., 2022). Each component may play a unique role in immune regulation. Thus, we conducted untargeted metabolic analysis of fecal samples from mice. *L. reuteri* treatment not only modulated the composition of intestinal microbiota but also increased the production of flavonoid compounds in the intestine, notably luteolin. In this study, we explored the potential medicinal value of luteolin, and compared with single-component drugs, the combination of *L. reuteri* metabolites may produce synergistic effects.

Luteolin, a flavonoid found in various plants such as vegetables, medicinal herbs, and fruits (Imran et al., 2019), has been shown to possess anti-allergic activity (Khan, 2014; Ueda et al., 2002). As a member of the flavonoid family, luteolin exhibits anti-inflammatory functions that may have clinical applications for patients with cancer, neurobehavioral disorders, or intestinal inflammation (Franza et al., 2021). Flavonoids interact with the gut microbiota by modulating its composition, thereby serving as precursors for the fermentation into additional bioactive compounds with anti-inflammatory properties (Loo et al., 2023).

LO treatment significantly reduced colon injury, inhibited the inflammatory response, and altered the structure and composition of the gut microbiota in ulcerative colitis rats (Li et al., 2021). Dong et al. (2021) discovered that LO effectively alleviated inflammation and restored the Th1/Th2 imbalance in AR rats by modulating the TLR4/NF-κB signaling pathway. In our study, Luteolin’s immunomodulatory properties may alleviate AR symptoms by balancing Th1/Th2 and Treg/Th17 cells. It can also modulate immune function by inhibiting the MAPK/STAT3 signaling pathway.

A study suggested that LO restored the balance between Treg and Th17 cells in a mouse model, where the levels of OVA-sIgE, IL-17A, and ROR*γ*t were elevated, while IL-10 and Foxp3 levels were reduced (Yang et al., 2023). A comprehensive review article explored the latest research on luteolin’s role as a natural immunomodulator, particularly focusing on its influence on inflammatory signaling mechanisms, including its impact on NF-κB, MAPK, and JAK/STAT pathways (Hussain et al., 2023). LO also exhibited a therapeutic effect on neutrophilic asthma by suppressing the secretion of IL-36γ, thereby modulating MAPK signaling pathways (Qiao et al., 2023). Network pharmacology showed that 251 luteolin against osteosarcoma targets and 8 hub targets including AKT1, ALB, CASP3, IL6, JUN, STAT3, TNF, and VEGFA (Huang et al., 2023).

The STAT3 signaling pathway is closely associated with the development of Th17 and Treg cells (Piao et al., 2020). Santana et al. (2019) showed sakuranetin inhibited of MAPK and STAT3-SOCS3 to attenuate chronic allergic airway inflammation in mice. Weixiong Chen et al. showed She-Chuang-Si-Wu-Tang alleviates inflammation and itching symptoms in a psoriasis mouse model by regulating the Th17/IL-17 axis via the STAT3/MAPK pathways (Chen et al., 2024). Our study demonstrated that LO treatment effectively reduced the p-p38/p38 and p-STAT3/STAT3 protein expression ratios. This suggests that microbially metabolized luteolin may influence immune function by balancing Th17 and Treg cells and regulating the MAPK/STAT3 signaling pathway.

The oral administration of *L. reuteri* can regulate the intestinal microecology, so that *L. reuteri* and its metabolites can indirectly act on the nasal mucosal immune system. The cross-organ regulation approach breaks the previous limitation of local treatment of nasal cavity, treats the whole body as a unified immune whole, and provides an innovative strategy for the treatment of complex diseases associated with multiple organs. *L. reuteri* can modulate the immune response, reshape gut microflora composition. We found that *Prevotellaceae_UCG_001* and *Odoribacter* were positively correlated with IL-4, IL-5, IL-13 cytokines, whereas a positive correlation between the *Ligilactobacillus* and IFN-γ cytokines. The increase in specific microbial abundance, and the alteration of intestinal microflora structure or metabolism may be related to the Th2 response. The negative correlation between reduced microbial abundance and Th2 response warrants further investigation. This could provide more effective personalized medicine treatments for specific microbial imbalances or metabolic dysfunction.

The mechanisms by which gut microorganisms and their metabolites exert their effects need further exploration, which may reveal more effective therapeutic strategies through metabolite interactions. *L. reuteri* may activate signaling pathways by increasing luteolin expression, inhibiting the aberrant Th2-type immune response. Though direct evidence is lacking, luteolin’s properties provide a plausible mechanism. Luteolin’s immunomodulatory properties suggest a mechanism for alleviating AR symptoms by restoring the Th1/Th2 and Treg/Th17 cell balance. Moreover, luteolin has the potential to modulate immune function by inhibiting MAPK/STAT3 signaling pathway.

We will further delve into the pharmacological mechanisms of luteolin in the future, providing evidence that *L. reuteri* treatment and its flavonoid compounds may be effective alternative strategies for alleviating AR. Future research should aim to investigate various metabolites to comprehensively understand their combined interactions. Additionally, animal and clinical studies should further evaluate the effects of probiotics and their metabolites on intestinal microflora to provide a more holistic view. Moreover, the findings from this study highlight the potential of probiotics as a complementary or alternative treatment for allergies, warranting further investigations into their long-term safety and efficacy in human subjects.

## Data Availability

The datasets presented in this study can be found in online repositories. The names of the repository/repositories and accession number(s) can be found in the article/supplementary material.
